# Editorial: Unmet Needs in Dystonia

**DOI:** 10.3389/fneur.2017.00197

**Published:** 2017-05-09

**Authors:** Alberto Albanese

**Affiliations:** ^1^Department of Neurology, Humanitas Research Hospital, Rozzano, Milano, Italy; ^2^Department of Neurology, Catholic University, Milan, Italy

**Keywords:** dystonia, network, cooperation, lumping, splitting

**The Editorial on the Research Topic**

**Unmet Needs in Dystonia**

The need for a cooperation on dystonia at the European level has been pursued for years. This movement disorder has been extensively studied in Europe, where important advances in knowledge have repeatedly taken place. The pioneering clinical physiologic approach led by David Marsden has deeply characterized European research on dystonia and more recently the clinical and genetic complexity of dystonia has been clarified.

In 2011, the European Cooperation in Science and Technology (COST) office approved a European Concerted Research Action designated as COST Action BM1101: European network for the study of dystonia syndromes. The original applicants were from 18 European countries, but later increased to encompass 24 European countries and the United States. By the end of the Action, in 2015, the countries involved were: Belgium, Bulgaria, Croatia, Denmark, France, Germany, Greece, Hungary, Ireland, Israel, Italy, Latvia, Republic of Macedonia, Netherlands, Norway, Poland, Portugal, Romania, Serbia, Slovakia, Slovenia, Spain, Sweden, and United Kingdom. Clinicians and researchers from these countries have interacted on research and clinical activities during the 4-year duration of the Action. The European Dystonia Federation (now Dystonia Europe) has been a fundamental partner and has also served as administrative grant holder.

The European dystonia players have been deeply engaged by this concerted activity, and our research and personal ties have been reinforced along time. The Action has involved industry partners in additions to patients, doctors, and researchers. This research topic is the Action final publication, following a final conference held on October 2015 at the campus of Humanitas Research Hospital in Rozzano. The title “Unmet needs in dystonia” was proposed by Kailash Bathia and adopted by the Steering committee to signify the complexity of this area of knowledge that is still characterized by more questions than answers. We consider that this leitmotiv represents well the current state-of-art of dystonia.

The common theme of this publication is lumping vs splitting. How many dystonia syndromes exist clinically, genetically, neurophysiologically, etc.?

The first chapter in this Research Topic, by Albanese, is a clinical and historical review ending with a proposal on the new diagnostic criteria for dystonia syndromes. The descriptions of dystonia from post-medieval ages till now are reported. This exercise accounts for innovative clinical observations and scholar thinking. The second chapter by Verbeek and Gasser reviews the genetic heterogeneity of dystonia syndromes. Whole-exome sequencing and genome-wide association studies have allowed the discovery of novel genes and risk factors for dystonia. How to combine clinical and genetic heterogeneity is a matter for future research.

The third chapter by Valls-Sole and Defazio is an update on blepharospasm. Phenomenology and electrophysiology are reviewed for this simple, yet complex, focal dystonia. A chapter by Contarino et al. reviews the complex management of cervical dystonia, the most common focal dystonia syndrome. In this area, we expect several innovations, including treatment of non-motor features and functional neurosurgery. Evidence-based recommendations for the treatment of cervical dystonia with botulinum toxin are reviewed in the fifth chapter. Cervical dystonia is an area where we expect most innovation in management with botulinum neurotoxins. Injection of deep cervical muscles, usage of ultrasound, and EMG combined are rewriting current clinical practice. The next chapter by Lin and Nardocci reviews the complex issue of childhood dystonia that is often combined with other movement disorders or neurodevelopmental issues. Finally, the needs and requirements of modern biobanks, an issue extensively discussed within the COST Action, are reviewed by Lohmann et al.

We hope that these contributions will be helpful to clinicians, researchers, and patients. Altogether they offer a glimpse on dystonia that is not available elsewhere.

This effort (and all the activities covered by the COST Acton) have been possible thanks to the dedication of Alistair Newton and Monika Benson (from Dystonia Europe), who deserve all our thanks. Their long-standing friendship under the name of dystonia and their stubborn dedication turned so many common initiatives, including this COST Action, into a success. The author also wish to thank the COST Office (now COST Association) and particularly Inga Dadeshidze and Jeannette Nchung Oru, who assisted us as Science and Administrative Officers.

## Author Contributions

The author confirms being the sole contributor of this work and approved it for publication.

## Conflict of Interest Statement

The author declares that the research was conducted in the absence of any commercial or financial relationships that could be construed as a potential conflict of interest.

